# Chondrocyte Apoptosis in the Pathogenesis of Osteoarthritis

**DOI:** 10.3390/ijms161125943

**Published:** 2015-10-30

**Authors:** Hyun Sook Hwang, Hyun Ah Kim

**Affiliations:** 1 Division of Rheumatology, Department of Internal Medicine, Hallym University Sacred Heart Hospital, Kyunggi 431-060, Korea; wazzup@hallym.ac.kr; 2 Institute for Skeletal Aging, Hallym University, Chunchon 200-702, Korea

**Keywords:** osteoarthritis, chondrocyte, apoptosis, caspase, mitochondria, chondroptosis, autophagy, endoplasmic reticulum stress, cartilage

## Abstract

Apoptosis is a highly-regulated, active process of cell death involved in development, homeostasis and aging. Dysregulation of apoptosis leads to pathological states, such as cancer, developmental anomalies and degenerative diseases. Osteoarthritis (OA), the most common chronic joint disease in the elderly population, is characterized by progressive destruction of articular cartilage, resulting in significant disability. Because articular cartilage depends solely on its resident cells, the chondrocytes, for the maintenance of extracellular matrix, the compromising of chondrocyte function and survival would lead to the failure of the articular cartilage. The role of subchondral bone in the maintenance of proper cartilage matrix has been suggested as well, and it has been proposed that both articular cartilage and subchondral bone interact with each other in the maintenance of articular integrity and physiology. Some investigators include both articular cartilage and subchondral bone as targets for repairing joint degeneration. In late-stage OA, the cartilage becomes hypocellular, often accompanied by lacunar emptying, which has been considered as evidence that chondrocyte death is a central feature in OA progression. Apoptosis clearly occurs in osteoarthritic cartilage; however, the relative contribution of chondrocyte apoptosis in the pathogenesis of OA is difficult to evaluate, and contradictory reports exist on the rate of apoptotic chondrocytes in osteoarthritic cartilage. It is not clear whether chondrocyte apoptosis is the inducer of cartilage degeneration or a byproduct of cartilage destruction. Chondrocyte death and matrix loss may form a vicious cycle, with the progression of one aggravating the other, and the literature reveals that there is a definite correlation between the degree of cartilage damage and chondrocyte apoptosis. Because current treatments for OA act only on symptoms and do not prevent or cure OA, chondrocyte apoptosis would be a valid target to modulate cartilage degeneration.

## 1. Physiological and Pathological Role of Apoptosis

The regulation of cell death and the subsequent post-mortem change is an essential process of healthy living. Programmed cell death, called apoptosis, which was first identified by Kerr [1], is a highly-regulated pathway that involves specific sets of intracellular signals and genes. Dysregulation of apoptosis leads to pathological states, such as cancer, developmental anomalies and degenerative diseases. During apoptosis, cells show morphological characteristics, such as chromatin condensation, DNA fragmentation, cell shrinkage, plasma membrane blebbing and the formation of apoptotic bodies [2].

Apoptosis plays a critical role in maintaining the homeostasis of various tissues in the adult human body, as well as regulating normal embryonic development. After tissue injury, a variety of cells, including neutrophils, macrophages and lymphocytes, are accumulated in the injury site to initiate tissue repair, and after the healing process is finished, the surplus of accumulated cells are eliminated by programmed cell death to prevent excessive inflammation and resulting tissue damage [3]. In addition, apoptosis is responsible for the elimination of germ cells during the process of oogenesis and post-ovulation [4] and the post-lactational involution of the mammary gland [5].

Dysregulation of apoptosis, thus, is related to a diversity of diseases, including developmental defects, autoimmune disease, neurodegeneration and cancer. Cancer cells manifest various alterations of the apoptosis pathway, conferring them resistance to apoptosis. Disrupted balance of pro- and anti-apoptotic proteins by underexpression and/or overexpression and dysregulated expression of microRNA, which regulates oncogenes or tumor suppressor genes, were observed in cancer cells [6,7,8], resulting in overproliferation and/or dysfunctional removal of cancer cells [9,10]. Fas and/or TNF-related apoptosis-inducing ligand (TRAIL) receptors, members of tumor necrosis factor (TNF) receptor superfamily, are highly upregulated in many carcinomas, such as hepatocellular, ovarian, renal, colorectal and pancreatic cancers [11]. On the other hand, acquired immune deficiency syndrome is caused by excessive apoptosis of T cells that results from internalization of human immunodeficiency virus into T cells via the CD4 receptor and increased expression of the Fas receptor [12]. Apoptosis is also responsible for neurodegenerative diseases, including Alzheimer’s disease, Parkinson’s disease (PD) and Huntington’s disease, where impaired functions of mitochondria, a pivotal player in the intrinsic pathway of apoptosis, increase the levels of reactive oxygen species (ROS), perturbing ATP production, membrane potential, permeability transition pore activation and calcium uptake [13]. Increased levels of caspase-1, -3 and -8 were observed in a 1-methyl-4-phenyl-1,2,3,6-tetrahydropyridine (MPTP)-induced mouse model and in PD and Alzheimer’s disease patients’ brains [14].

## 2. Mediators of Apoptosis

### 2.1. Caspases

Caspases, key molecules in the processes of apoptosis, are known to regulate not only apoptosis, but also non-apoptotic functions, including cell proliferation, differentiation and migration. Caspases contain an amino-terminal prodomain, a p20 large subunit with the active site cysteine, and a carboxy-terminal p10 small subunit and are synthesized as inactive zymogen and activated by proteolytic cleavages. At present, 14 mammalian caspases have been identified, and they are classified into initiator caspases (caspases-2, -8, -9 and -10), effector caspases (caspases-3, -6 and -7) and the inflammatory caspases (caspases-1, -4, -5, -11, -12 and -13) (Table 1) [15,16]. Initiator caspases have long prodomains that interact with the motifs, such as the death effector domain (DED) or the caspase recruitment domain (CARD) present in the upstream adaptor proteins. Effector caspases, which have a short prodomain, are activated after cleavage by upstream initiator caspases [17]. Activated effector caspases cleave multiple cellular substrates, including cytokeratins, poly(ADP-ribose) polymerase (PARP), the plasma membrane cytoskeletal proteins α fodrin and nuclear mitotic apparatus protein (NuMA). Caspase-3, the most important executioner caspase, cleaves inhibitor of caspase-activated DNase (ICAD), which inhibits caspase-activated DNase (CAD) that functions as endonuclease. The inflammatory caspases are involved in the pathogen-induced cell death mechanism and not in classical apoptosis pathways [16,18]. Caspase-1 is activated by inflammasomes, which are assembled by the NOD-like receptor (NLR) or pyrin and the HIN domain-containing protein (PYHIN) family member and promotes activation of pro-IL-1β and pro-IL-18 cytokines, which play pro-inflammatory roles [19]. Caspase-11 is activated by cytosolic lipopolysaccharide (LPS) receptors via the CARD domain [20].

Because caspases are activated by irreversible proteolysis, caspase activation is tightly regulated through caspase inhibitors, caspase degradation and decoy inhibitors [16]. Inhibitor of apoptosis proteins (IAPs), known as endogenous proteins that regulate the activity of initiator and effector caspases, suppress apoptosis in response to death receptor activation and growth factor depletion. IAPs are listed in Table 1 [21]. The IAPs contain one or three baculovirus IAP repeat (BIR) domains at the N-terminus and the RING-zinc finger domain, which possesses E3 ubiquitin ligase activity, at the C-terminus. C-IAP1 and c-IAP2 distinctly possess a CARD domain that mediates oligomerization with other CARD-containing proteins. For example, XIAP interacts with and inhibits caspase-9 via its BIR3 domain and caspase-3 and -7 via its BIR2 domain [22]. In addition, because many IAPs contain RING and ubiquitin-associated domains responsible for ubiquitination, cellular levels of IAPs can be regulated via the proteasome [23]. Caspases can be inhibited by decoy proteins that possess nonfunctional catalytic domains, structurally similar to caspase prodomains, and prevent caspase from being activated within the activation platform [16]. Cellular FLICE-inhibitory protein (c-FLIP), including long form of c-FLIP long (c-FLIP_L_) and a short form of c-FLIP (c-FLIP_S_), contains two DEDs incorporated into the death-inducing signaling complex (DISC) complex through DED-DED interactions and interferes with activation of caspase-8 and -10. However, another study suggested that c-FLIP_L_ induces auto-catalysis of pro-caspase-8 at the DISC and functions as a pro-apoptotic inducer [24].

**Table 1:** Mediators implicated in the apoptosis pathway.

Protein Name	Description
1. Caspases family	
Initiator caspases	- Activated by dimerization via DED or CARD in adaptor proteins
Caspase-2	
Caspase-8	
Caspase-9	
Caspase-10	
Executioner caspases	- Activated by proteolysis
Caspase-3	
Caspase-6	
Caspase-7	
Inflammatory caspases	- Activated by dimerization
Caspase-1	
Caspase-4	
Caspase-5	
2. Caspase inhibitors	
Inhibitors of apoptosis (IAP)	- Containing one or three tandem BIR domains, RING-zinc finger domain, or CARD domain
X-linked inhibitor of apoptosis	
Cellular IAP1/Human IAP2	
Cellular IAP2/Human IAP1	
Testis-specific IAP	
BIR-containing ubiquitin conjugating enzyme	
Survivin	
Livin	
cellular FLICE- inhibitory protein (c-FLIP)	- Containing two DED - Recruited to the DISC through DED–DED interactions
c-FLIP_L_ (c-FLIP long)	
c-FLIP_S_ (c-FLIP short)	
3. Bcl-2 family	
Anti-apoptotic members	- Containing BH1, BH2, BH3, BH4, and TM domains
Bcl-2	
Bcl-XL	
Bcl-W	
Mcl-1	
A1	
Multi-domain pro-apoptotic members	- Containing BH1, BH2, BH3, and TM domains
Bax	
Bak	
BH3-only pro-apoptotic members	- Containing BH3 domain or BH3 and TM domains
Bid	
Bad	
Bim	
Bik	

### 2.2. B-Cell Chronic Lymphocytic Leukemia (CLL)/Lymphoma 2 (Bcl-2) Family Proteins

The Bcl-2 family members control mitochondrial apoptotic signaling by modulating mitochondrial membrane permeability. The *Bcl-2* gene was first identified as a proto-oncogene, and 20 members of the Bcl-2 family have been found in mammals. The Bcl-2-related protein is characterized by the presence of one or more of four conserved Bcl-2 homology (BH1–BH4) domains. Based on their structure and function, Bcl-2 family members are classified into anti-apoptotic or pro-survival members, multi-domain pro-apoptotic members and BH3-only pro-apoptotic members. The anti-apoptotic members, including Bcl-2, Bcl-Xl, Bcl-w, Mcl-1 and A1, contain four domains of BH1, BH2, BH3 and BH4 and a carboxy-terminal transmembrane domain (TM) that allow them to integrate into the outer mitochondrial membrane, endoplasmic reticulum (ER) and the nuclear envelope. The multi-domain pro-apoptotic members of the Bcl-2 family, such as Bax and Bak, which have BH1, BH2, BH3 and TM domains, are responsible for disruption of organellar membranes and induction of caspase activation, while the BH3-only pro-apoptotic members containing only the BH3 domain include Bid, Bad, Bim and Bik and function as initiators of apoptosis [25]. Bcl-2 family proteins can be readily heterodimerized by protein-protein interactions between pro- and anti-apoptotic Bcl-2 family members, which determine whether cell survival or the apoptosis signal proceed [25,26]. For example, when a BH3-only protein interacts with a pro-survival protein, the activity of the latter is neutralized, and apoptosis is promoted [25,26]. Structural studies on anti-apoptotic members of the Bcl-2 family revealed that the presence of the hydrophobic helix on the molecules is required for their binding to their cognate pro-apoptotic partners, which promotes pro-survival activity [27,28,29].

## 3. Apoptosis Signaling Pathways

The mechanism of apoptosis are divided into the intrinsic or mitochondrial pathway, which is induced by intracellular signals, and the extrinsic or death receptor pathway, which is triggered by the extracellular signals, including activation of the death receptor family [30]. However, the two apoptosis pathways are interconnected through the mitochondria.

**Figure 1:**
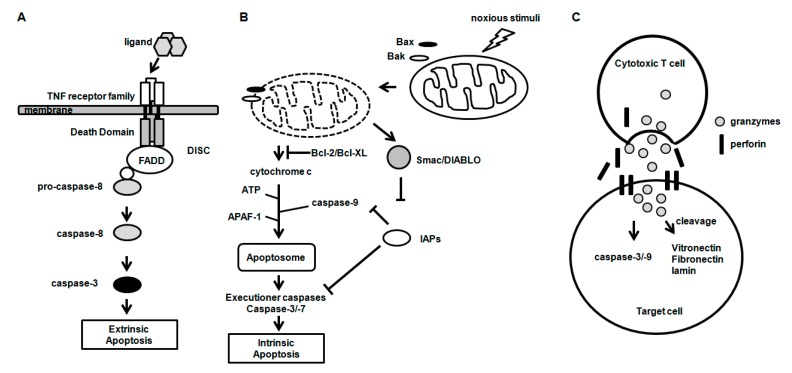
Three major mechanisms of apoptosis. (**A**) Intrinsic and (**B**) extrinsic apoptosis pathway; and (**C**) Granzyme-mediated apoptosis pathway.

The death receptors, including Fas (CD95/APO-1), TNFR and TRAIL receptor 1 and 2, belong to the TNF receptor superfamily characterized by the presence of a death domain (DD), a cytosolic domain and a cysteine-rich extracellular domain [30,31]. In the extrinsic apoptosis pathway, the death receptors perceive the extracellular apoptosis signal by binding their respective ligands. Binding of a ligand (FasL, TNF-α and TRAIL) to the death receptors forms a complex called DISC, which recruits the adaptor protein Fas-associated death domain (FADD) and procaspase-8 by interacting via the DD (Figure 1A). Caspase-8 activated by auto-processing at DISC activates the downstream effector caspases, including caspase-3, which, in turn, activates various target molecules independently of mitochondria, leading to apoptosis. However, in certain cell types, apoptotic signaling by active caspase-8 was known to be insufficient for activation of downstream caspases, such as caspase-3 and -7. In these cells, the Bid cleaved by caspase-8 (tBid) translocates to the mitochondria and activates the mitochondrial apoptosis pathway.

The intrinsic apoptosis pathway is triggered by non-receptor-mediated stimuli, when the absence of growth factors, hormones and cytokines fails to sustain the survival signal or when noxious stimuli, such as radiation, toxins, hypoxia and free radicals, activate the death pathway (Figure 1B) [30]. Mitochondrial outer membrane permeabilization (MOMP), a key event in the intrinsic pathway, leads to the release of mitochondrial intermembrane proteins, such as cytochrome c (cyt c), the serine protease Omi/high temperature requirement protein A2 (HtRA2) and the second mitochondria-derived activator of caspase/direct IAP binding protein (Smac/DIABLO) into the cytosol [32]. Cytosolic Bax and Bak proteins translocate to mitochondria and turn into the Bak–Bax oligomers within the outer mitochondrial membrane, leading to the release of cyt c into the cytosol. Released cyt c forms the apoptosome complex together with apoptosis protease activating factor-1 (Apaf-1), cyt c and cofactor dATP/ATP. Procaspase-9 is recruited to the apoptosome via interaction with the CARD of Apaf-1. Consequently, caspase-9 is activated by proteolytic cleavage and activates pro-caspase-3 and -7, the executioner caspases [17,33,34]. In addition, released Smac/DIABLO activates caspases by inhibiting IAP, which mediates caspase-9 inhibition [35]. In the pancreatic β cell line, a cytokine mixture of IL-1β, IFN-γ and TNF-α induced mitochondrial dysfunction with a significant loss of mitochondrial membrane potential and upregulated caspase-3 activity, revealing the possibility that proinflammatory factors might induce apoptosis by the mitochondrial pathway [36].

Besides the intrinsic and extrinsic pathways, granzymes, granule-secreted enzymes, are involved in apoptosis through caspase-dependent and -independent mechanisms [37]. In granzyme-mediated apoptosis, granzyme B and perforin are released from the granules of cytotoxic cells (Figure 1C). Granzyme B, which has access to the cytoplasm of target cells by a perforin-mediated endocytosis process, cleaves a number of substrates, including vitronectin, fibronectin and laminin [38], and initiates apoptosis through caspase-dependent and -independent pathways.

## 4. The Pathogenesis of Osteoarthritis (OA)

OA is the most common chronic arthritis in the elderly, characterized by gradual degradation of articular cartilage, synovial inflammation and pain, resulting in significant disability. Multiple factors contribute to the degradation of cartilage in OA, by either directly or indirectly regulating the anabolic and catabolic pathways of the cartilage matrix. Articular cartilage is a highly specialized tissue consisting of an extracellular matrix that is synthesized solely by the sparsely-distributed resident cells, the chondrocytes. The survival of the chondrocytes is, thus, important for the maintenance of proper cartilage matrix, and the compromising of chondrocyte function and survival would lead to the failure of the articular cartilage. The role of subchondral bone in the maintenance of proper cartilage matrix has been suggested, as well, and it has been proposed that both articular cartilage and subchondral bone interact with each other in the maintenance of articular integrity and physiology. Some transcriptional factors exert functions in both tissues: for example, the Cbfa1 transcriptional factor acts for osteoblast differentiation, as well as chondrocyte maturation, while Cbfβ is essential to maintain cortical thickness through stabilizing Runx2 in osteoblasts and to differentiate chondrocytes through stabilizing Runx2 and Runx3 proteins in the cartilage [39,40]. Thus, some investigators include both articular cartilage and subchondral bone as targets for repairing joint degeneration [41,42,43,44,45].

The degeneration of cartilage might be caused by the reduced number of chondrocytes in the aged human articular cartilage, which fails to regenerate and remodel the cartilage appropriately [46]. In the early stage of OA, chondrocytes get larger and assemble groups or clumps of cells, sometimes consisting of 50 or more cells per group, which are called “chondrocyte clones”. This phenomenon is regarded as the evidence of the metabolic activity of cartilage and the replicating ability of chondrocytes in OA [47]. On the contrary, despite the active proliferation of chondrocytes, the glycosaminoglycan (GAG) content in the matrix appears decreased compared to a non-OA control matrix, suggesting that the level of compensation is suboptimal [48]. In late stage of OA, the cartilage exhibits a central feature of chondrocyte death, including hypocellularity and lacunar emptying.

## 5. Implication of Chondrocyte Apoptosis in OA Pathogenesis

Contradictory reports exist on the rate of apoptotic chondrocytes in osteoarthritic cartilage, ranging from clearly less than 1% up to about 20% [49,50]. In the calcified cartilage layer, a higher number of empty lacunae are observed compared to other cartilage layers, and a decreased number of living chondrocytes in the calcified layer may play a deleterious role, in particular in the more advanced-stage disease, because it represents the major portion of the remaining cartilage matrix [51]. Since apoptosis is usually a quick process, a high rate of apoptosis in cartilage would theoretically result in matrix degradation within a very short period of time, which is not compatible with the chronic course of OA. Altogether, apoptosis clearly occurs in osteoarthritic cartilage; the relative contribution of chondrocyte apoptosis in the pathogenesis of OA is difficult to assess, and thus, it would be an over-simplification to state that apoptotic chondrocyte death is the main pathogenetic mechanism in OA.

In contrast to necrosis, apoptosis is characterized by the absence of the uncontrolled release of lysosomal enzymes, which is largely possible by the phagocytosis of the apoptotic bodies. With the absence of phagocytes, such as tissue-resident macrophages, and the presence of the extracellular matrix, which prevents phagocytosis of apoptotic remnants by other cells, secondary necrosis would be inevitable in articular cartilage. On the other hand, a recent study showed CD163-positive chondrocytes in the cartilage mid-zone of temporomandibular joints and knee from rats, with an increase in their number with enhanced phagocytic activity in type II collagen positive chondrocytes isolated from the degenerated cartilage, suggesting a role in eliminating degraded tissues [52].

The term “chondroptosis” was coined to describe the type of cell death that is commonly observed in chondrocytes, reflecting the fact that chondrocytes do not undergo apoptosis in a classical manner. Chondroptosis is characterized by the following features: marked increase of the amount of Golgi, ER and primary lysosomes, the presence of autophagic vacuoles and the extensive blebbing or extrusion of cellular material into the lacunae and peri-lacunar matrix [51].

Chondrocyte death, be it apoptotic, necrotic or chondroptotic, may result in the same outcome in view of the failure to appropriately maintain the structure of articular cartilage. Most eukaryotic cells attach to neighboring cells or to the extracellular matrix for survival, a phenomenon called anchorage dependence. Because the damage of proteoglycan and the collagen network in the cartilage matrix is the dominant pathologic features of cartilage degradation, it is plausible that chondrocyte anchorage to the extracellular matrix is disturbed to a significant degree to cause chondrocyte apoptosis. *In vitro* enzymatic treatment of cartilage explant cultures with high-purity collagenase, which specifically cleaves type II collagen, significantly induces chondrocyte apoptosis, underlining the importance of the intact collagen fibril to sustain chondrocyte survival [53]. Blocking or knocking down of integrin, which provides a link between the extracellular matrix and the cytoskeleton, causes chondrocyte apoptosis in chick sternal cartilage organ culture and induces severe cartilage degradation accompanied by GAG depletion, increments in MMP-2, MMP-3 and apoptotic chondrocytes [54,55]. To seek the connection between cartilage matrix degradation and chondrocyte apoptosis, it is difficult to confirm whether cell disintegration is primary or secondary to the destruction of the cartilage matrix. An animal OA study elucidating the role of specific inhibitors of apoptosis on its effect on the protection from cartilage degeneration would be needed. Chondrocyte death and matrix loss may form a vicious cycle, with the progression of one aggravating the other. Although the literature reveals that there is a correlation between the degree of cartilage damage and chondrocyte apoptosis, it is also of note that in 40–60-year-old donors’ cartilage, there are unusually high numbers of apoptotic chondrocytes, also in macroscopically normal cartilage [56].

## 6. The Mechanism of Chondrocyte Apoptosis

**Figure 2:**
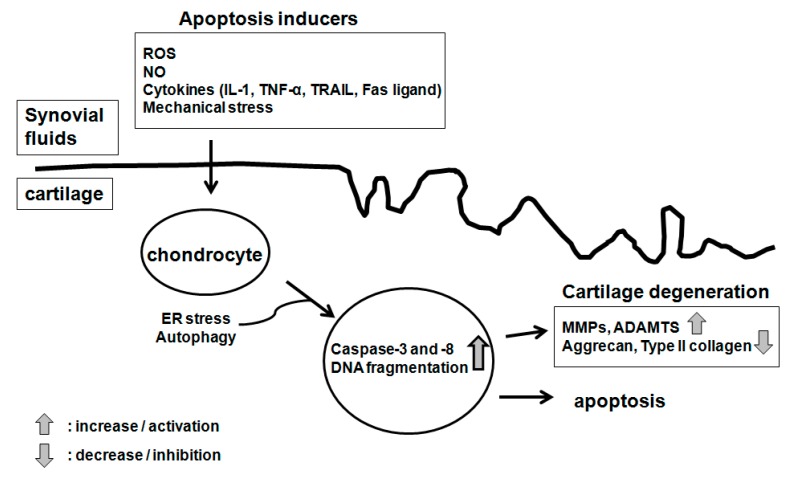
Inducers of chondrocyte apoptosis.

Cultured chondrocytes go through apoptosis in response to diverse stimuli, including serum starvation, treatment with the Fas ligand or anti-Fas/CD95 antibodies, the NO donor sodium nitroprusside (SNP), staurosporine/dihydrocytochalasin B and ceramide [49,50,57,58,59,60,61] (Figure 2). In the case of assessing these *in vitro* results, however, caution must be taken with regard to the pathophysiological relation, such as whether the apoptosis-inducing stimuli can also be pathologically implicated as inducers of arthritis. Furthermore, it should be kept in mind that *in vitro* responses can be largely dependent on the species, the condition of the donor (whether young or aged and whether osteoarthritic or normal) and the culture condition (monolayer, explant or alginate beads).

### 6.1. Molecular Inducers of Chondrocyte Apoptosis

OA cartilage produces a large amount of NO, which suppresses the cartilage matrix synthesis, and enhances MMP activity [62]. Although treatment with NO donors consistently leads to apoptosis in cultured chondrocytes, the precise role of NO in the induction of chondrocyte death is currently questioned, because the increased level of the iNOS did not lead to chondrocyte death [57,63]. Chondrocyte apoptosis induced with NO warrants cautious interpretation, since studies mostly utilized chemicals, such as SNP or *S*-nitroso-*N*-acetyl-l-penicillamine (SNAP). Previous reports demonstrate that peroxynitrite, a reaction product of NO and superoxide anions, is the main player in chondrocyte apoptosis, with a low level of ROS inducing apoptosis in the presence of NO and a high level of ROS inducing necrosis [64,65]. On the other hand, chondrocyte death involves calpains, calcium-dependent cysteine proteases and peroxynitrite-induced mitochondrial dysfunction in chondrocytes, leading to caspase-independent apoptosis [66].

IL-1, the major cytokine mediating cartilage degradation, is reported to decrease chondrocyte viability in some animal species [67]. However, IL-1 alone fails to induce human chondrocyte death and even protects cultured chondrocytes against Fas-mediated apoptosis [57,68]. Although TNF-α-induced chondrocyte apoptosis is debated, TRAIL, which functions critically in tumor surveillance by controlling apoptosis, directly causes chondrocyte apoptosis [69]. Normal human articular chondrocytes were known to express the TRAIL receptors modulating apoptosis (DR4 and DR5), and cartilage obtained from experimentally-induced OA rats showed an increased expression of TRAIL and DR4, implicating a role for TRAIL-induced chondrocyte apoptosis in the pathogenesis of OA [70]. The release of intracellular contents from apoptotic cells, as well as local production of inflammatory mediators might play a further role in cartilage degradation. For example, chondrocytes obtained from intra-articular fractures show higher expressions of pro-apoptotic factors and chemoattractive receptor ChemR23, suggesting that sustained chondrocyte death, related to the persistently high levels of proinflammatory factors, could augment the degradation of cartilage tissue [71].

Because human OA chondrocytes express the Fas receptor, a classical receptor for the apoptosis-inducing pathway engaging the FAS ligand, its role in chondrocyte apoptosis was postulated. Although the anti-Fas antibody induces apoptotic body formation and increases TUNEL-positive chondrocytes, there has been still controversy over whether Fas-mediated apoptosis is directly induced in cultured chondrocytes [59]. There is an inherent barrier to Fas receptor engagement on chondrocytes in articular cartilage, because chondrocytes are not in close proximity to each other.

### 6.2. Modulation of Mitochondrial Activity

Mitochondria play a key role in cellular function and survival, and oxidative stress and disrupted mitochondrial respiration were reported to promote cell death and degeneration. Chondrocytes from OA cartilage showed a significant decrease of mitochondrial electron transport chain activity in complex II and III, in addition to a decrease in the mitochondrial membrane potential (Δψ_m_), which is related to mitochondrial swelling and damage of the outer mitochondrial membrane [72]. A comparative proteomics study showed significant changes in mitochondrial proteins involved in energy production, maintenance of mitochondrial membrane integrity and free radical detoxification, as well as a decrease in mitochondrial superoxide dismutase (SOD) levels and an increase of intracellular ROS generation in OA chondrocytes [73]. Depolarization of the mitochondria leads to the release of apoptotic factors, such as cytochrome *c*, apoptosis-inducing factor and capase-9, from the mitochondrial intermembrane space to the cytoplasm and leads to apoptosis, which is augmented by intracellular reactive oxygen species (ROS) [74]. Bcl-2, an anti-apoptotic protein, is found in the outer mitochondrial membrane and prevents mitochondrial permeabilization and release of cytochrome c. Chondrocytes expressing antisense Bcl-2 showed increased apoptosis, even under the condition of 10% serum, as compared to wild-type cells, while chondrocytes overexpressing Bcl-2 were protected from apoptosis induced by both serum withdrawal and retinoic acid treatment [75]. This result shows that modulation of the mitochondrial respiratory chain activity could be a therapeutic target to modulate chondrocyte apoptosis in OA.

### 6.3. MicroRNA and Chondrocyte Apoptosis

MicroRNAs (miRNA) are endogenously-synthesized small oligonucleotides (~22 bp), which regulate post-transcriptional gene expression. miRNAs control multiple genes, both by suppressing translation and accelerating mRNA degradation, and up to a third of all human genes contains putative miRNA recognition elements [76]. The pathogenetic role of miRNAs in the development of OA was suggested in a study that showed that miR-140^−/−^ mice had proteoglycan loss and fibrillation of articular cartilage, while transgenic (TG) mice overexpressing miR-140 in cartilage were protected from antigen-induced arthritis [77]. miR-146a is induced in response to many microbial components and pro-inflammatory cytokines, including IFN-α and IL-1β, and is expressed in OA cartilage [78,79]. miR-146a has gained interest, as a negative correlation between its expression and MMP-13 expression level was observed, and it was found to regulate key signaling intermediates of the pro-inflammatory Toll-like receptor (TLR)-MyD88 pathway [78,79]. In a recent report, miR-146a was found to increase by mechanical pressure injury, and its upregulation induced apoptosis in human chondrocytes through the targeted inhibition of Smad4 in cartilage [80]. In addition, IL-1β responsive miR-146a is overexpressed in a surgically-induced joint instability OA model in rat, accompanied by upregulation of VEGF and downregulation of Smad4 [81], suggesting that miR-146a has a dual role as both an inducer of cartilage degradation and of chondrocyte apoptosis. The regulation of multiple genes by miRNA and the demonstration of the differential expression of miRNA in animal cells and tissues has led to the supposition that their mechanism of action is theoretically similar to that of transcription factors [82]. The field of miRNA research in OA is still in progress, and it may have exciting therapeutic potential.

### 6.4. Chondrocyte Senescence and Apoptosis

Aging is characterized by a progressive loss of function and physiological integrity, resulting from genomic and epigenetic change, cellular senescence and altered cellular interaction [83]. Advanced age is the greatest risk factor related to OA, and it is presumed that chondrocyte senescence induced by a variety of harmful biomechanical factors induces stress and causes irreversible damage, leading to cell death. A recent study shows that there is a markedly-reduced cellularity in articular cartilage with aging, and a moderate to strong positive correlation exists between the degree of cartilage damage and chondrocyte apoptosis [56]. Extracellular inorganic pyrophosphate (PPi) and PPi-generating nucleoside triphosphate pyrophosphohydrolase (NTPPPH) are upregulated in articular cartilage from aged individuals, which is associated with chondrocalcinosis in OA cartilage. Transfection of NTPPPH isoenzyme markedly conferred enhanced apoptosis to cultured chondrocytic knee meniscal cells, demonstrating a direct connection between aging, apoptosis and cartilage degeneration [84]. Apoptotic bodies isolated from NO-treated chondrocytes include alkaline phosphatase and NTPPPH activities and precipitated calcium, suggesting that chondrocyte-derived apoptotic bodies may directly affect cartilage calcification seen in aging and OA [85]. Aged chondrocytes are more susceptible to cell death induced by an NO donor as revealed by an *in vitro* study [86]. It was suggested that increased oxidative stress is the culprit mechanism, as the susceptibility was associated with a higher ratio of oxidized glutathione to reduced glutathione. Aging is associated with low-grade chronic systemic inflammation characterized by an imbalance between inflammatory and anti-inflammatory pathways [87]. Autophagy decreases in aging cell, and as a result, proteins are aggregated, misfolded proteins are accumulated and dysfunctional mitochondria are formed, all culminating in oxidative stress. In addition, pro-inflammatory cytokines provoke inflammatory responses and inhibit autophagy, leading to an increase in chondrocyte apoptosis [88]. Aging has been implicated in elevating extra-cellular matrix stiffness, and advanced glycation end-products (AGEs), in particular, are considered as a main player in driving nonenzymatic collagen cross-linking [89]. Elevated levels of AGE induced by ribose treatment *in vitro* significantly increased the elastic moduli of collagen matrices, together with reduced deposition of proteoglycan, increased MMP expression and activity and reduced expression of cartilage ECM molecules in embedded chondrocytes [90]. In addition, *in vivo* intracellular AGE deposition induced by intra-articular injection of AGE precursors resulted in ER stress and apoptosis in chondrocytes and degraded articular cartilage [91]. There is evidence of an age-related decrease in the chondrocyte response to growth factors. The mitogenic response of human chondrocytes to IGF-1 was decreased with donor age [92]. Oxidative stress suppressed IGF-1-stimulated Akt phosphorylation, while upregulating phosphorylation of ERK, and these effects were more pronounced in cells from older donors, in line with an age-related decline in the response of human chondrocytes to IGF-1 and OP-1 [93].

### 6.5. Autophagy, Endoplasmic Reticulum (ER) Stress and Chondrocyte Apoptosis

Autophagy is a cellular homeostatic mechanism involving the catabolic process of energy recycling in eukaryotic cells [94]. It is induced by a wide range of stimuli, such as metabolic stress and oxygen depletion, and plays a key role in the regulation of cell death and inflammation [88,95]. Dysregulation of autophagy has been associated with diverse disorders, including cardiomyopathy, neurodegeneration and abnormal skeletal development [96]. Mammalian target of rapamycin (mTOR), a serine/threonine protein kinase, is a major negative regulator of autophagy. The function or regulatory mechanism of mTOR is dependent on whether it forms a complex with Raptor (mTORC1) or Rictor (mTORC2). Akt directly phosphorylates and activates mTOR, while mTOR negatively regulates phosphatidyl inositol-3-kinase (PI3K)/Akt activity and activates protein synthesis and ribosome biogenesis through phosphorylation of S6 kinase and the eIF-4E binding protein [97,98,99,100,101]. Autophagy has been shown to engage in a complex cross-talk with apoptosis, initiated by shared upstream signals, sometimes resulting in simultaneous autophagy and apoptosis [102]. Apoptotic and autophagic response machineries share common pathways: Beclin 1, the major component of autophagosome formation, has been identified originally as a Bcl-2-interacting protein, possessing a Bcl-2 homology 3 domain (BH3-only), which is necessary for its binding to the BH3 receptor domain of Bcl-2 or Bcl-X_L_. While Bcl-2 inhibits the autophagic function of Beclin 1, disruption of the Beclin 1/Bcl-2 complex by mutation of the BH3-only domain within Beclin 1 leads to the stimulation of autophagy [103,104]. Beclin 1 and class III PI3K, two components of the autophagy-inducing complex, are direct substrates of caspases [105]. Flip (FLICE inhibitory protein), an inhibitor of death receptor-mediated apoptosis, inhibits autophagy through a direct interaction with crucial autophagy-relevant proteins, such as LC3 and Atg3 [106]. In addition to the role of apoptosis regulatory factors in the modulation of autophagy, the apoptosis-associated cleavage of Beclin 1, Atg5 and Atg4D can eliminate cytoprotective autophagy and form protein fragments, promoting the execution of the apoptotic program [107].

Previous reports have shown that LC3-II, an autophagy marker, is upregulated in the chondrocytes and cartilage of patients with OA, indicating that there is a relationship between autophagy and cartilage degeneration [98,101]. On the other hand, decreased autophagy was found in OA articular cartilage and in an animal OA model, and autophagy activation protected chondrocytes from death, revealing the protective role of autophagy in chondrocyte death and cartilage degeneration [108,109,110]. Furthermore, rapamycin, an inhibitor of mTOR, reduced cartilage degeneration in bovine and human cartilage explants through the activation of LC3 and suppressed glucocorticoid-stimulated chondrocyte death [108,109,111]. Mitochondrial dysfunction induced by treatment with oligomycin was associated with increased production of reactive oxygen species and cell death, as well as a decrease of autophagy activation. Pretreatment with rapamycin and torin 1 before oligomycin significantly protected against mitochondrial dysfunction, highlighting the role of autophagy as a critical protective mechanism against mitochondrial dysfunction [112]. Autophagy may promote chondrocyte survival or death depending on donor age and the sort of autophagy inducer, because rapamycin-induced autophagy inhibited the build-up of sub-diploid cells in young chondrocytes, and inhibition of autophagy by 3-MA, an inhibitor of PI3K type III, downregulated TNF-α-induced chondrocyte death through the suppression of both autophagy and autophagy-induced apoptosis, suggesting a death-promoting role for autophagy in the pathogenesis of OA, as well [113].

In eukaryotic cells, the membrane and secreted proteins are folded and assembled in the ER before delivery to other cellular compartments or the extracellular milieu. Poorly-folded proteins are retained and targeted for degradation, and such an ER protein quality control mechanism can be overwhelmed by a variety of insults, such as hypoxia or infection, resulting in ER stress [114]. When the cells fail to successfully adjust to ER stress or to reestablish homeostasis, the levels of ER protein-folding enzymes and chaperones are increased, leading to the activation of unfolded protein response (UPR), which triggers a set of cell death programs. The induction of ER chaperones, such as the 78-kDa glucose-regulated protein (Grp78), enhances folding activity in the ER, while the activation of the ubiquitin-proteasome system in the cytosol degrades unfolded or misfolded proteins [115]. ER stress-induced apoptotic events are mediated by transcriptional activation of the gene for C/EBP homologous protein (Chop), which directly regulates death effectors, such as Bcl-2 and Bim [116,117]. ER stress and UPR signaling affect the pathogenesis of numerous diseases, including neurodegeneration, cancer, diabetes and inflammation [118].

The expression of Grp78 and Bcl-2-associated athanogene-1 (bag-1), markers of ER stress, was upregulated in articular cartilage obtained from advanced OA patients, suggesting the role of ER stress in the pathogenesis of OA [119]. ER stress induces the expression of ER stress-related genes and activation of apoptosis in chondrocytes of chop-knockout mice and human OA cartilage [114,120]. ER stress-induced genes, including XBP1S and IRE1α, were reported to affect ER stress-mediated apoptosis in chondrocytes [121,122,123]. ER stress-induced chondrocytes showed decreased expression of aggrecan and type II collagen, as well as increased expression of pro-catabolic factors, suggesting its role as a mediator not only of apoptosis, but also of cartilage degeneration [121,122,124].

A recent study showed that over-activation of autophagy significantly reduces the UPR in the neonatal hypoxia-ischemia model, highlighting the relevance of the cross-talk between ER and the autophagy machinery and possible therapeutic targeting of the relevant molecular mechanism in the treatment of OA [125].

### 6.6. Other Forms of Programmed Cell Death in Chondrocytes

Previous reports suggested that other types of cell death mechanisms are implicated in the programmed cell death of chondrocytes. Granzyme B, an apoptosis-inducing factor, is expressed in arthritic cartilage and chondrocytes and, when introduced into chondrocytes, induces apoptosis in a dose-dependent manner. Chondrocytes expressed the surface antigens of NK cells and showed cytotoxicity against K562 cells, indicating that chondrocytes have NK cell-like activity associated with innate immunity [126,127].

A recent study showed that complement effectors, and specifically, the membrane attack complex (MAC)-controlled arm of the complement, play an important role in the pathogenesis of OA in different mouse models [128]. MAC led to the production of inflammatory and degradative molecules in cultured chondrocytes and co-localized with MMP-13 in human OA cartilage. Although extensive accumulation of MAC causes cell lysis and necrotic cell death, many of the MAC-encircled chondrocytes in OA cartilage were morphologically intact, excluding its role in chondrocyte apoptosis [129]. Instead, MAC increased MMPs, ADAMTSs and cyclooxygenase 2, as well as complement effectors in chondrocytes, implicating synergistic role of complement produced in synovium to amplify pathogenic complement signaling in OA.

In a study determining the occurrence of chondrocyte apoptosis during the progression of OA in the STR/ort mouse model, chondrocyte death with many of the morphological features of typical apoptosis was detected and correlated with the development of OA [130]. However, caspases were not detected, suggesting a caspase-independent mechanism of cell death. Caspase-independent chondrocyte death was further defined in peroxynitrite-induced death, which involved calcium-dependent cysteine proteases (calpains), and in chondrocyte necroptosis induced by the retention of D469del-COMP, a mutated cartilage oligomeric matrix protein gene (COMP) implicated in pseudoachondroplasia [66,131].

## 7. Targeting Apoptosis in OA Treatment

Current treatments for OA are focused on alleviating the symptoms, rather than preventing or curing OA, and patients with OA are mostly treated with paracetamol or non-steroidal anti-inflammatory drugs (NSAIDs). As OA is a slowly-progressing, indolent disease, effective agents that virtually prevent the destruction of articular cartilage with minimal toxicity have been sought after. Chondrocyte apoptosis would be a valid target to modulate cartilage degeneration, because the loss of chondrocyte vitality is a significant hallmark of human OA.

However, pharmacological and biological inhibitors of apoptosis may have potential harmful systemic effects, such as carcinogenesis, warranting discretion in their pursuit. The caspase inhibitor was the most studied among all of the apoptosis regulators in OA. *In vitro* studies exhibited that the non-selective caspase inhibitor, Z-VAD-FMK, and the caspase-3 selective inhibitor, Z-DEVD-FMK, significantly suppressed chondrocyte apoptosis induced by collagenase, okadaic acid camptothecin, TNF-α and staurosporine treatment and reduced serum conditions [132,133,134]. Cartilage from dogs induced to OA with anterior cruciate ligament transaction (ACLT) showed that chondrocyte apoptosis was significantly reduced when treated *in vitro* with inhibitors of caspases, such as Z-DEVD-FMK or Z-LEHD-FMK (caspase-9 inhibitor) [135]. The caspase inhibitor was found to inhibit chondrocyte apoptosis induced by a mechanical injury consisting of static stress of 14 MPa for 500 ms in human cartilage explants [136]. In a rabbit ACLT transection model of OA, intraarticular injections of the pan-caspase inhibitor Z-VAD-FMK significantly suppressed cartilage degradation accompanied by reduced expression of PARP p85 and active caspase 3 in chondrocytes [137]. The caspase inhibitor may be effective for diseases where tissue damage is limited to cell death, such as post-traumatic arthritis, in which there is a time window after injury when cartilage cells can be salvaged or protected from the progression of cell death [138].

While there is a paucity of data showing the direct effect of apoptosis inhibition in the treatment of OA, numerous studies report the protective role of putative therapeutic agents for OA in chondrocyte apoptosis. For example, intra-articular IL-1 receptor antagonist (IL-1 ra, anakinra) in a Lewis rat ACLT model increased lubricin expression and lubricin synovial fluid lavage concentrations, while it decreased caspase-3 positive chondrocytes [139]. Intraarticular hyaluronan (HA) injections reduced OA severity and the number of apoptotic chondrocytes in a rabbit ACLT model, while diacerein, a drug with IL-1 inhibitory activity, reduced cartilage degradation in an animal OA model, as well as DNA fragmentation and death of chondrocytes in *ex vivo* OA knee cartilage obtained from a dog ACLT model [140,141,142]. On the other hand, the pharmacological doses of glucosamine HCl, the most widely-used nutraceutical for the treatment of OA, was found to induce a decline in the metabolic activity of bovine chondrocytes, resulting in cell death [143].

## 8. Conclusions

Apoptosis offers a multitude of potential targets for pharmacological treatment to protect articular cartilage in OA. The recent advance in the elucidation of the apoptosis mechanism has inspired renewed interest in this field. The cross-talk between autophagy, ER stress and apoptosis, as well as the newly-recognized role of miRNA in the regulation of apoptosis would offer novel insights for therapeutic interventions. Since it was shown in a guinea pig OA model that the increase of chondrocyte apoptosis and cartilage degradation was preceded by a progressive thickening and stiffening of the subchondral bone plate, modulation of subchondral bone modeling should be considered as a necessary future aspect of investigation [144]. The extent to which apoptotic signaling pathways can be effectively and safely modified to prevent or suppress chondrocyte apoptosis in OA, however, still remains to be fully elucidated.
